# Homemade: building the structure of the neurogenic niche

**DOI:** 10.3389/fcell.2023.1275963

**Published:** 2023-12-01

**Authors:** Ghanim Fajish Valamparamban, Pauline Spéder

**Affiliations:** Institut Pasteur, Université Paris Cité, CNRS UMR3738, Structure and Signals in the Neurogenic Niche, Paris, France

**Keywords:** neural stem cell (NSC), niche, architecture, development, mouse, zebrafish, *Drosophila*

## Abstract

Neural stem/progenitor cells live in an intricate cellular environment, the neurogenic niche, which supports their function and enables neurogenesis. The niche is made of a diversity of cell types, including neurons, glia and the vasculature, which are able to signal to and are structurally organised around neural stem/progenitor cells. While the focus has been on how individual cell types signal to and influence the behaviour of neural stem/progenitor cells, very little is actually known on how the niche is assembled during development from multiple cellular origins, and on the role of the resulting topology on these cells. This review proposes to draw a state-of-the art picture of this emerging field of research, with the aim to expose our knowledge on niche architecture and formation from different animal models (mouse, zebrafish and fruit fly). We will span its multiple aspects, from the existence and importance of local, adhesive interactions to the potential emergence of larger-scale topological properties through the careful assembly of diverse cellular and acellular components.

## 1 Introduction

Our central nervous system (CNS) is an exquisitely complex organ which allows us to live in and interact with our environment by receiving, integrating and sending information (Purves et al., 2019). It relies on the functions of highly specialized cells, the neurons, which form intricate cellular connections and ultimately networks to handle these exchanges. Alongside, glial cells (astrocytes and oligodendrocytes) are key players ensuring structural support and insulation of neuronal fibers, as well as a diversity of regulatory roles. Both cell types must be produced and integrated in time and space to assemble into a functional community.

The complete process of generating new functional neurons is called neurogenesis and encompasses cell division, migration, differentiation, maturation, and integration into circuits (Binder et al., 2009; Squire et al., 2009; Purves et al., 2019). Similarly, gliogenesis produces functional glial cells. In this review, we will use neuro/gliogenesis when we do not wish to specifically restrict or distinguish between the two.

Developmental neuro/gliogenesis supports the formation of a functional nervous system in animals, whether centralized or diffuse (Bayraktar et al., 2014; Paridaen and Huttner, 2014; Holguera and Desplan, 2018; Villalba et al., 2021). It is unsurprisingly high, taking place throughout the tissue at a sustained pace, and often extends for some time after birth/hatching. Throughout this period, an extensive range of neuronal and glial subtypes is generated.

At later stages (from infancy to adulthood and into ageing), neuro/gliogenesis still happens in many organisms, with variable extents of outputs and locations, defying a long-held notion that there was little capacity for *de novo* production of neurons and glia outside of development (Colucci-D’Amato et al., 2006; Bond et al., 2015). Evidence of adult neuro/gliogenesis has now been reported for teleost fishes (Byrd and Brunjes, 2001), cephalopods (Bertapelle et al., 2017), amphibians (Bernocchi et al., 1990) and reptiles (Marchioro et al., 2005), song birds (Goldman and Nottebohm, 1983), rats (Altman and Das, 1965) and mice (Reynolds and Weiss, 1992), non-human primates (Gould et al., 1998) and humans (Eriksson et al., 1998) -a non-extensive list (for an illuminating comparative view, see (Lindsey and Tropepe, 2006)).

Both in a developing or adult nervous system (we will focus here on centralized systems), decades of study have shown that neuro/gliogenesis is initiated and supported by cells endowed with remarkable properties, the neural stem cells.

## 2 Building a nervous system from stem cells

Tissue stem cells are responsible for the formation and maintenance of the tissue/organ they reside in. To do so, they balance self-renewal with differentiation to maintain their pool while generating specialized cells. They can also shuttle between proliferation and quiescence, a reversible, mitotically dormant phase. A precise regulation of stem cell identity, fate and features (stemness; self-renewal; survival; proliferation; differentiation) is thus crucial to the health of their tissue of residence (for an inspiring discussion on the definition of stem cell, see (Potten and Loeffler, 1990)).

The stem cells of the CNS are called neural stem cells (NSCs). NSCs are traditionally defined as multipotent progenitors able to self-renew through their lifetime to generate the diversity of neuron and glial cells which makes up the nervous system ((Temple, 2012) and see (Breunig et al., 2011) for an historical perspective). Thus far, NSCs have been discovered in many species, and their identification keeps increasing thanks to advancements in single-cell sequencing technologies. Best developed experimental models comprise rodents (mouse, and rat to a lesser extent), fishes (zebrafish, and medaka and killifish to a lesser extent), amphibians (*Xenopus*), birds (especially songbirds) and invertebrates (mainly *Drosophila melanogaster*). Newer or on the rise models include octopus (Styfhals et al., 2022), hydra (Galliot and Quiquand, 2011) and axolotl (Lust et al., 2022).

During development, NSCs, from ectodermal origin, are found throughout the CNS. They actively proliferate, mixing expansion, through symmetric division, with differentiation, through asymmetric division (Schmidt et al., 2013; Paridaen and Huttner, 2014; Mira and Morante, 2020), to ensure that the correct amount of progeny is generated while preserving their pool. Asymmetric division can be directly neurogliogenic, but often rather produce progenitors with more restricted potential, and which further divide to generate neurons and/or glia.

In adults, NSCs are also present and active to support neuro/gliogenesis, whose extent varies between species. In most teleost fishes, for example, the CNS keeps growing along with the skull, and NSCs are found in extensive locations, providing constant neuro/gliogenesis (reviewed in (Zupanc, 2021)). Conversely, in mammals, NSCs are found in restricted locations, where they provide both basal and adaptive neuro/gliogenesis (reviewed in (Gage, 2019; Obernier and Alvarez-Buylla, 2019). In humans in particular, adult neuro/gliogenesis also happens in restricted places, and potentially declining with age, with its extent still debated (Ghibaudi and Bonfanti, 2022). Nevertheless, a common trait of adult neuro/gliogenesis is the balance between proliferative and quiescent states to provide homeostatic control of the NSC population and/or long-term NSC maintenance and protection. Quiescence is proposed to exist in multiple shades, from deep to shallow, with an active on-going research to find discriminating markers and crucial regulators (Urbán et al., 2019).

It is worth noting that the frontier between NSCs and neural progenitors can sometimes be hazy, and during development, the overarching term of neural stem/progenitor cells (NSPCs) is often used. In the developing mouse for example, NSCs *stricto sensu* are the neuroepithelial cells and the radial glia, yet at the single cell level these exist in a range of identity, fate and time restriction with no differentiating markers. The advent of single-cell technologies has indeed brought forward the idea that cell identity might be a continuum rather than a series of discrete states (Marcy and Raineteau, 2019). Live-imaging also revealed an unexpected range of individual cellular behaviour and fate, with some restricted progenitors (transient amplifying) able of prolonged symmetric divisions while stem cells (radial glia) can directly differentiate into two neurons (Pilz et al., 2018). Earlier studies already showed that most radial glia primarily produce neurons, while some are specialized in generating glial cells (Malatesta et al., 2000; Pinto et al., 2008). This raised questions about their multipotency and suggested that many radial glia may already have lineage restrictions, making them better suited to a progenitor classification. An interesting discussion on stemness, potency and generally cell individuality in the CNS can be found in (Petrik et al., 2022).

## 3 What is a neurogenic niche?

### 3.1 Emergence and evolution of the concept of stem cell niche

While a well-defined genetic program instructs stem cell behaviour during development and homeostasis, they are also exposed and sensitive to the extrinsic signals coming from their cellular microenvironment. Indeed, tissue stem cells are not isolated but rather embedded within a complex and specific neighbourhood made up by cellular as well as acellular cues. From this observation rose the concept of the stem cell niche.

Shortly after the discovery of tissue stem cells in the hematopoietic system, and pioneering work on their potential regulation by their cellular context (Lord et al., 1975; Dexter et al., 1977), Schofield indeed proposed that stem cells were found in and regulated by a unique localized microenvironment he termed stem cell niche (Schofield, 1978). This initial definition was drawn from the ecological field and also within the experimental context of the mammalian haemopoietic stem cells, one of the flourishing models at the time. Stem cells were believed to lose their characteristics like self-renewal or ability to differentiate into other cell types if taken away from their residency site. Conversely, a cell could become stem cell if transplanted in such niche. Other examples of niche, for different stem cells and in different biological systems, followed to confirm the importance of such microenvironment in stem cell maintenance. In mammals, tissue stem cells and their niches were also uncovered in the intestine (Cheng and Leblond, 1974), skin (Cotsarelis et al., 1990) and brain (Eriksson et al., 1998; Doetsch et al., 1999). Experimental evidence from *Caenorhabditis elegans* identified stem cells in the germline in constant communication with nearby somatic cells to maintain their features (Kimble and White, 1981). Similarly, a landmark paper showed in *Drosophila* *melanogaster* that somatic cells in the female ovariole organise into a cellular microenvironment that maintains and controls germline stem cells (Xie and Spradling, 2000). Stem cell niches were also identified in plants, starting with the shoot apical meristem of *Arabidopsis thaliana* (Clark et al., 1997).

Yet, the identification over the years of more and more stem cell types, both during developmental and adult stages, and in a diversity of organisms, slowly challenged the initial, strict definition of the stem cell niche (Lander et al., 2012). NSCs can keep their stemness and proliferate outside of their normal cellular microenvironment (Temple, 1989), forming neurospheres. Not every cell can become stem cell once implanted in a stem cell niche. The conversion of transplanted cells into tissue-specific stem cells might also not only depend on inductive properties of the niche, but rather be in combination with the plasticity/reprogramming potential of these cells themselves; for example, NSCs transplanted in irradiated mice produce a variety of blood cell types, acquiring an hematopoietic identity (Bjornson et al., 1999).

### 3.2 The stem cell niche nowadays

So, what defines a stem cell niche nowadays? Our opinion is that a combination of functional and spatial attributes could hold the answer.

First, we propose that the niche enables and supports the physiological roles of tissue stem cells. These roles can encompass survival and maintenance of stemness as initially proposed, but also finer, yet crucial, characteristics, such as the temporal generation of diverse progeny, cell fitness or the balance between proliferative and quiescent phases. As such, the underlying idea here is not that tissue stem cells cannot exist without a niche, but rather that they cannot perform their normal homeostatic program and response to perturbations.

Second, the niche retains a proximity element, encompassing cells, cell parts (*i.e.*, cytoplasmic extensions) and acellular components in direct contact or very close vicinity of the stem cells. As such, neighbouring stem cells can also be considered part of one stem cell’s niche, and a stem cell progeny is an automatic component, at least in the timescale following its generation. While we propose that the concept of a “distant” niche, regulating stem cells from afar, can also be brought in, it can be redefined as a special case in which even though the initial component might be remote, its acting element(s) (soluble molecule, cytoplasmic extensions) are able to reach the stem cells. As illustration, adult NSCs are regulated by the innervation of neurons from the distant hypothalamic region (Paul et al., 2017) and also by circulating factors produced from remote sources and carried by the blood (Horowitz et al., 2020) and the cerebro-spinal fluid (CSF) (Silva-Vargas et al., 2016). While the cell bodies generating this innervation and factors are far, their products are ultimately in contact with the stem cells. Linked to this proximity aspect is also the concept that the niche is not merely a collection of juxtaposed components but harbours a stereotypical architecture, organising stem cells spatially and poising them for specific interactions.

Third, the niche acts as a signalling hub for the stem cells. It can produce and exchange its own paracrine signals, be the local receiver of further cues, and it is also an interface and mediator of systemic and environmental cues. For instance, Paneth cells of the intestinal stem cell niche secrete crucial signalling molecules for stem cell function and protection (Cui et al., 2023). An extension of its signalling role is the capacity of the niche to coordinate signals, between different tissue compartments and with environmental cues. For instance, in the *Drosophila* testis, the niche coordinates responses to extrinsic stress signals like high temperature or toxin exposure by activating the JAK-STAT pathway in the germline stem cells, ensuring the appropriate production of sperm in changing conditions (Tulina and Matunis, 2001).

### 3.3 The neurogenic niche: definition and core components

The neurogenic niche is the niche of the NSCs, and of neural progenitors in a larger extent. It is predicted to co-exist with NSPCs, yet so far has only been properly characterized for few species and at given stages. From these different models, and in line with what we discussed above, a current, prevailing view of the neurogenic niche brings the proximity of cellular and molecular components with their ability to influence NSPC behaviour while conveying informational cues (Binder et al., 2009; Bjornsson et al., 2015; Llorente et al., 2022). The neurogenic niche is typically a supportive and specialized microenvironment in which NSPCs reside. A core set of common cellular components has also emerged from these models, comprising the NSPCs themselves, different types of neurons and glia, a local circulatory system and finally cells with some immune or cell clearance capacity. Moreover, a “distant” niche often exists as a secretory organ. Around this definition and features exist declinations in space, time, composition and roles depending on the species.

**FIGURE 1:**
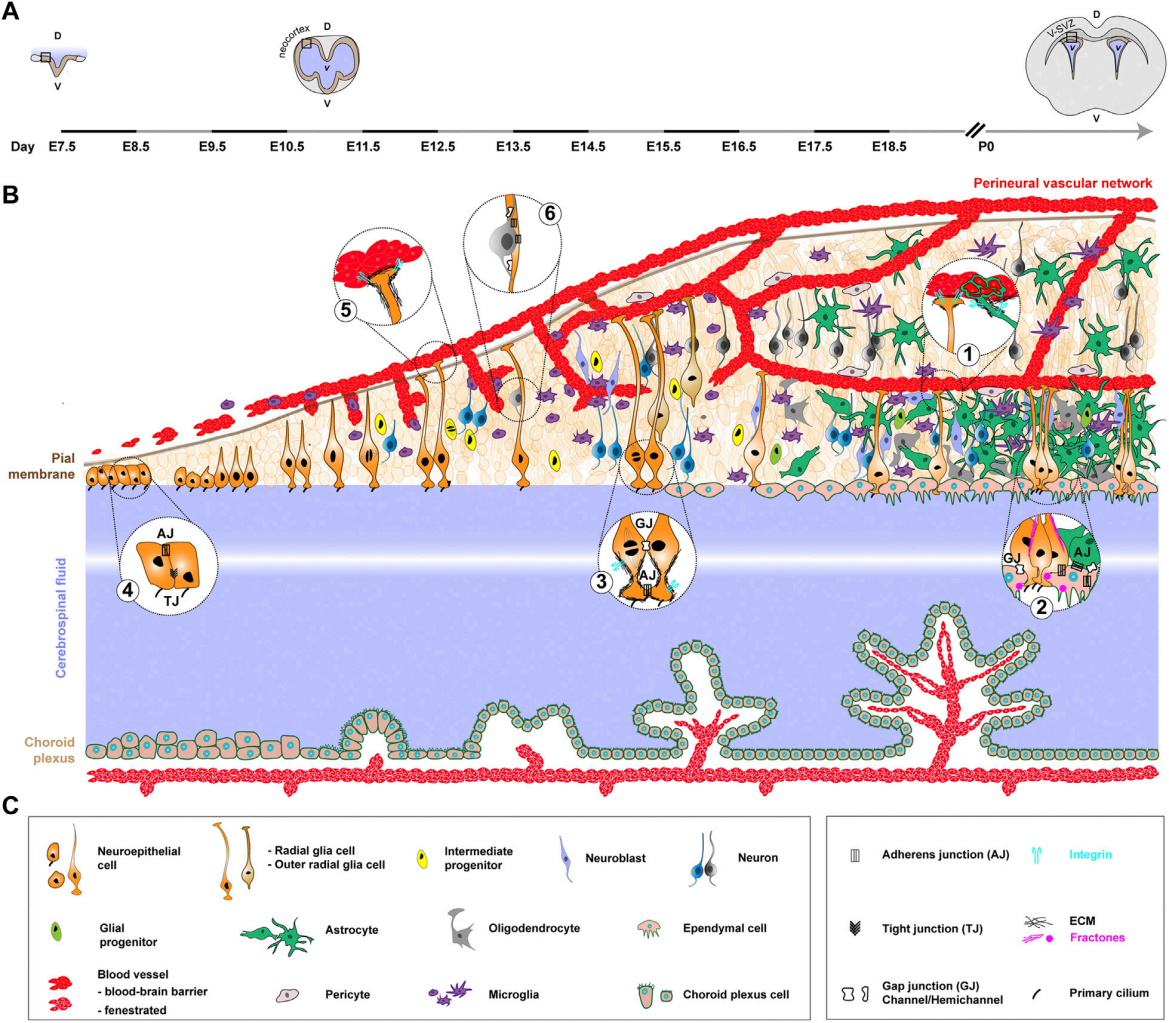
Formation of the neurogenic niche in the mouse, from early embryonic development to adulthood. This figure illustrates ventricular neurogenesis, which occurs along the wall of the CSF-filled ventricles. It serves as a model for understanding the core principles governing the formation and organization of the neurogenic niche in mice. It starts from the ventricular zone of the developing neocortex in embryonic stages and reaches the restricted location of the V-SVZ of the lateral walls (mature niche) by early postnatal period and then adulthood. **(A)**. Coronal view of the developing mouse CNS at different stages and associated timeline. The timeline, left to right, covers early embryonic development (embryonic day 7.5, E7.5) to early postnatal period. The stages depicted are neural groove (E7.5), closed and developed neural tube at the onset of neurogenesis (E11.5) and post-birth/young adult (P0, pups 0 h). The black rectangle highlights the zone represented in **(B)**. D, dorsal. V, ventral. *v*, ventricle. **(B)**. NSPCs mostly comprise the neuroepithelial cells and RGCs they convert into (orange), with also a contribution of outer RGCs (light brown). The apical processes of RGCs lie in the ventricular zone, whereas their elongated basal processes extend to the outer surface of the brain, connecting to the pial surface and blood vessels. NSPCs divide both symmetrically to amplify their pool and asymmetrically to give rise to intermediate neural (yellow) progenitors, further leading to the production of neuroblasts (light purple) and neurons (blue or grey, colour-coded to show temporal series). The basal process of RGCs guides newborn neurons to migrate away from the V-SVZ niche into the outer surface of the CNS to differentiate and function by forming connections. The neuronal subtypes which are produced, and their destinations, are different between embryonic and adult neurogenesis, yet the general principles of generation and migration are preserved. Late during embryonic development and during the early postnatal period, neural progenitors largely produce glial cells, including astrocytes (green) and oligodendrocytes (grey), either through the generation of glial progenitors (aniseed colour) or direct conversion. They also convert into ependymal cells (beige) which will separate the niche from the CSF (in light blue/purple, with a central white hue to represent greater distance). In parallel, three cell populations from external origin will contribute to the neurogenic niche. First, microglia (purple), generated from precursors in the yolk sac, enter the CNS during early development, where they will amplify and mature. A complex network of ventricular vasculature (red) is also built from sprouting and invading vessels coming from a peri-CNS plexus (perineural vascular network). Pericytes (light grey), from heterogeneous origin, are recruited to blood vessels to help forming the blood-brain barrier. Finally, the choroid plexus (beige), in continuity with the ependymal layer, is formed in remote locations from folding epithelial sheet, encloses blood vessels as they form and offers an example of a distant neurogenic niche. Bubbles contain magnification of specific structural features of the niche, completed by relevant adhesions and other molecular complexes (not depicted on the cellular timeline). **(C)**. Legends of the cellular and molecular features depicted in **(B)**.

**FIGURE 2:**
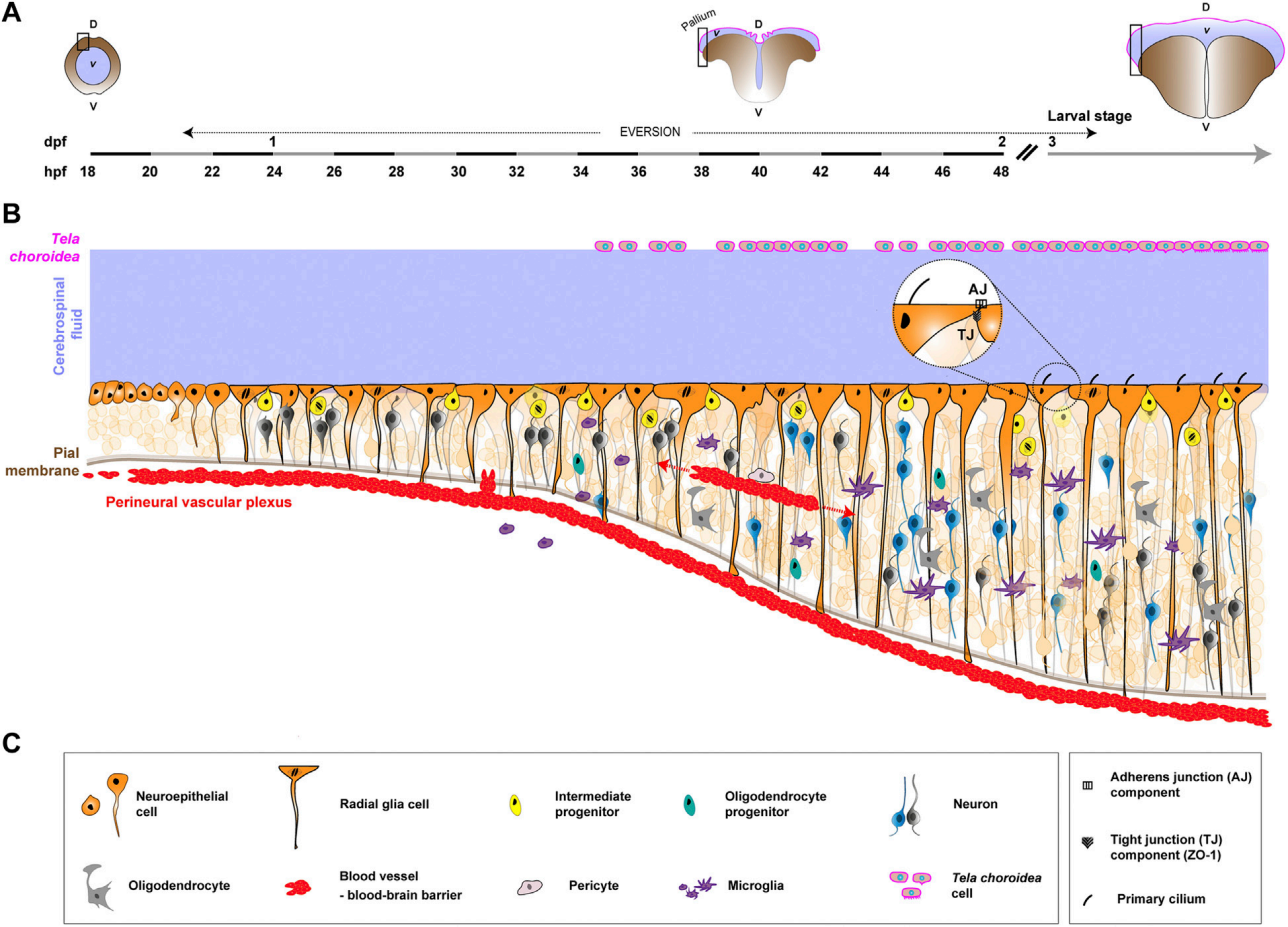
Formation of the neurogenic niche in the zebrafish pallium, from early embryonic development to larval/adulthood stage. Pallial neuro/gliogenesis happens in a continuous fashion from embryonic to adult stages and offers a straightforward link between developing and mature neurogenic niches. **(A)**. Coronal view of the developing zebrafish CNS at different stages and associated timeline. The timeline, left to right, covers early embryonic development (18 h post-fertilization, 18 hpf) into the larval stage (3 days post-fertilization, 3 dpf). The stages depicted are newly-formed neural tube (18–20 hpf), during telencephalon eversion (18 hpf to 5 dpf) and post-hatching/larval stage (from 3 dpf). The black rectangle highlights the zone represented in **(B)**. D, dorsal. V, ventral. *v*, ventricle. While a neural tube filled with CSF (light blue/purple) is originally formed, this canal structure is transient. The neuroepithelium undergoes eversion, exposing proliferative zones on the outer surface of the telencephalon. The *tela choroidea* (magenta line) covers the ventricle dorsally, encasing the CSF. **(B)**. Schematics of the formation of the neurogenic niche of the zebrafish pallium. Similarly to mammals, NSPCs (orange) are neuroepithelial cells and RGCs, successively. The later exhibit a radial, apico-basal polarity, with their basal process reaching the pial surface. Asymmetric cell divisions occur, with a Notch-driven signaling between daughter cells determining their fate and producing more restricted progenitors (yellow) which generate neurons (dark grey and blue) from 24 hpf. Some Oligo2^+^ progenitors (duck egg) give rise to oligodendrocytes (grey) around 30 hpf, which further mature and myelinate. External components also make the zebrafish neurogenic niche. First, microglia (purple), derived from the yolk sac, begin to colonize the CNS during mid-embryogenesis. They shift from an ameboid-like shape to a ramified morphology. Vascularization (red) starts with vasculogenesis and angiogenesis, forming the perineural vascular plexus around the brain and spinal cord. In different CNS regions, angiogenic endothelial tips sprout and connect to create a dense intraneural network. Blood-brain barrier function is established around 2.5–3 days post fertilization (dpf), coinciding with angiogenesis, and is supported by pericytes (light grey). Yet, most of the knowledge on CNS vascularization is derived from other regions, and much is left to understand in the pallium. Finally, the *tela choroidea* (magenta) covers the pallial sheet of radial glial cells in the zebrafish. Its morphogenetic events and role are little documented. **(C)**. Legends of the cellular and molecular features depicted in **(B)**.

**FIGURE 3:**
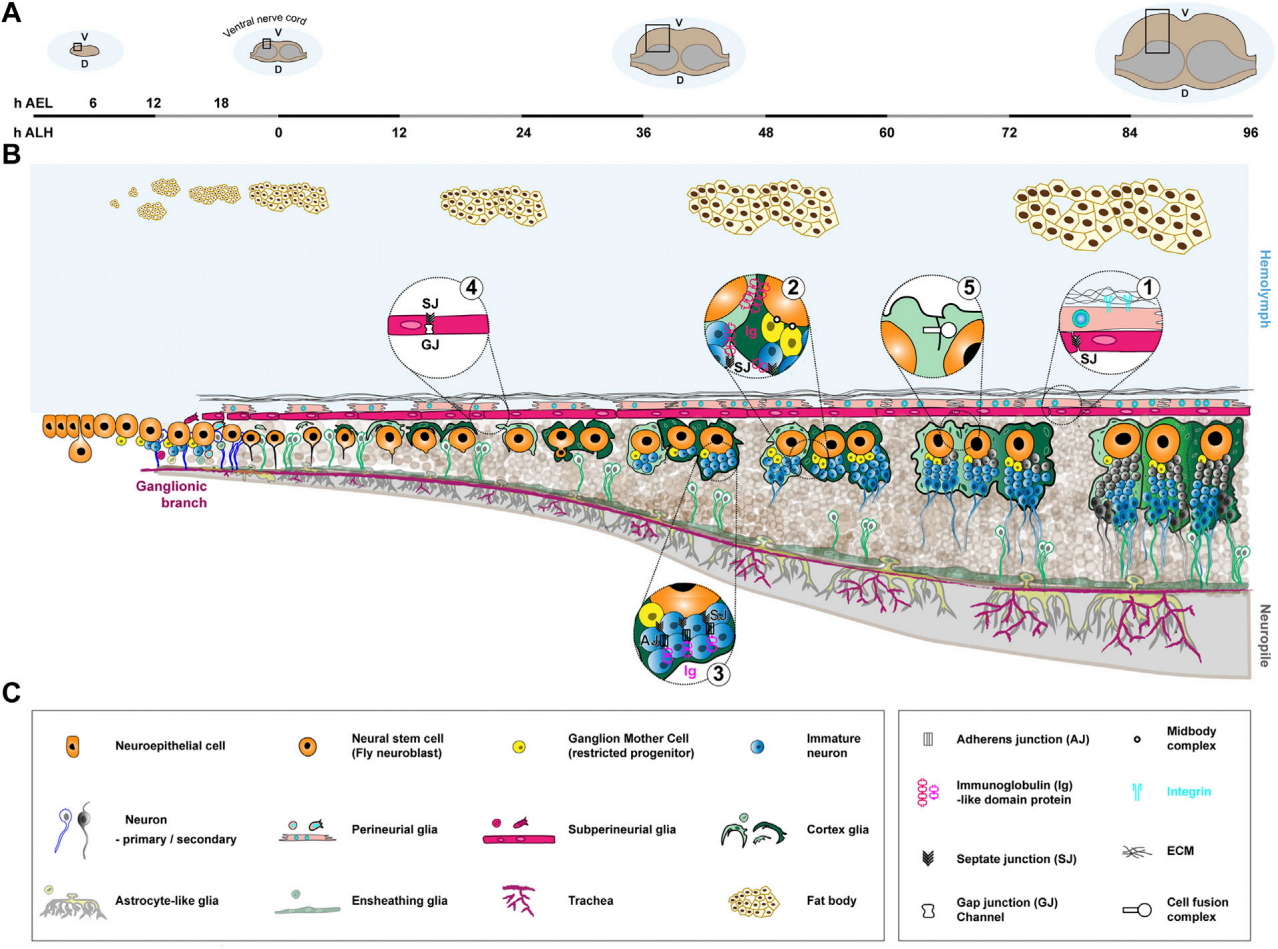
Formation of the neurogenic niche in the *Drosophila* larval ventral nerve cord. Neuro/gliogenesis in the ventral nerve cord is sustained by Type I NSCs from the embryonic stage to late larval (mature) stages, with a clear spatial and temporal continuity allowing to follow the formation of the corresponding neurogenic niche. **(A)**. Coronal view of the *Drosophila* CNS (ventral nerve cord) at different stages and associated timeline. The timeline, left to right, covers the embryonic (0–22 h after egg laying (AEL) at 25°C) and larval stages (0–96 h after larval hatching (ALH) at 25°C). The stages depicted are early embryo (6 h AEL), just hatched larva (0 h ALH), when NSCs have reactivated (36–48 h ALH) and at the end of larval stage, when NSCs prepare to exit the cell cycle (96 h ALH). The black rectangle highlights the zone represented in **(B)**. D, dorsal. V, ventral. **(B)**. Schematics of the formation of the neurogenic niche of the *Drosophila* ventral nerve cord. NSCs (orange) are generated by delamination from a neuroectodermal layer during embryogenesis and persist during the larval stage. They actively proliferate during embryogenesis, relying on asymmetric division to self-renew and generate a restricted precursor, the Ganglion Mother Cell (yellow), which will divide once more to give birth to two primary neurons (white body and blue outline). NSCs then enter a quiescence phase around 16 h AEL, which lasts until the beginning of larval stages. They then reactivate upon nutritional cues and divide asymmetrically to generate secondary neurons (blue and grey bodies and outlines). Gliogenesis mostly happens during embryogenesis, during which cortex glia (light and dark green) are born. Cortex glia are in close association with NSCs and progeny. They also encapsulate mature primary, embryonic (white body and green outline) and secondary (blue and grey bodies and green outlines) neurons. Cortex glia undergo morphogenetic processes during larval stages: expansion (growth, 0–36 h ALH), chamber formation around neural stem cell (24–48 h ALH), and extension (growth to adapt to lineage size, 48–96 h ALH). Cortex glia growth is sustained by different replicative strategies, making them polyploid (represented through bigger nuclei) and multinucleated (represented through smaller nuclei in higher number). Chamber formation involves the encasing of individual NSC and secondary lineage within a continuous layer of cortex glia membrane. Neurons from the same lineage remain together within a cortex glia chamber during maturation and extend fasciculated axons into the neuropile (light grey), where axons meet and synapses form. One cortex glia cell can encase several individual neural stem cell lineages, forming a regional mosaic structure (illustrated by different colours of cortex glia). In addition, cellular fusion occurs between cortex glia cells, leading to changing boundaries. The blood-brain barrier consists of the neural lamella (black mesh), perineurial glia (beige), and subperineurial glia (pink), and separates the CNS from the hemolymph (light blue). The ECM of the neural lamella is deposited early on and is remodeled during larval stages. Perineurial and subperineurial glia both are generated during embryogenesis and migrate to the CNS surface. Perineurial glia start as a squamous-like epithelium, then form a continuous monolayer between the neural lamella and subperineurial glia following growth mechanisms. The subperineurial glia already seal the barrier through septate junctions at late embryogenesis and undergo endoreplicative growth during larval stages to adapt to CNS development. Both ensheathing (green) and astrocyte-like (lime green) glia are positioned at the interface between cortex glia and the neuropile and regulate axonal and synaptic properties. They are generated during embryogenesis and grow during larval stage to accommodate CNS expansion. They have not been characterized as *bona fide* niche cells yet, and thus are represented in transparency. An external component of the niche is the tracheal system (dark pink), responsible for supplying oxygen. It originates from embryonic ectodermal cell clusters along the body, undergoing invagination and branching processes to send tracheal tracts to the different organs. Ganglionic branches target the ventral nerve cord around 10 h AEL and by the beginning of the larval stage form a rudimentary tracheal plexus which will grow and undergo branching throughout larval stages. Finally, the fat body (light yellow cells with brown outlines) is known to regulate NSC reactivation from quiescence and forms an example of a distant neurogenic niche. It starts as individual cell clusters during embryonic stages, which will divide, fuse and then further proliferate to perform diverse metabolic and immune functions. **(C)**. Legends of the cellular and molecular features depicted in **(B)**.

This review aims to discuss how such a neurogenic niche is built during development, specifically how its different components come together and interact to form its architecture, comparing the three current main systems for the study of neuro/gliogenesis: the mouse, the zebrafish and the fruit fly. This implies the choice of a well-defined neurogenic niche as a destination, what we will call here a mature niche. Before delving into niche formation, we will thus first introduce the composition and architecture of these selected mature niches. It will correspond to the adult ventricular-subventricular zone in the mouse (end of the timeline in Figure 1), the adult pallium in the zebrafish (end of the timeline in Figure 2) and the late larval ventral nerve cord in the fruit fly (end of the timeline in Figure 3). We will then describe how a rollercoaster of events during development generate these mature niches as a result of spatial and temporal continuity.

## 4 The mature neurogenic niche across species

### 4.1 Neurogenic niches in the adult mouse

The neurogenic niche was initially primarily studied, and as such defined, in the adult mouse (*Mus musculus*). It is owing to the restricted locations of *bona fide* NSCs in the adult, making it easier to delineate and describe their microenvironment. Two main neurogenic zones were identified in the adult mouse: the ventricular-subventricular zone of the lateral ventricle (V-SVZ), and the subgranular zone of the dentate gyrus of the hippocampus (SGZ). There, NSCs (named Type B cells in the SVZ (Doetsch et al., 1999) and Radial glia-like 1, RGL, in the SGZ (Eriksson et al., 1998)) exist mostly in a quiescent state (Type B cells can be quiescent for months (Obernier et al., 2018)), to which some awake to produce more restricted progenitor cells (transient amplifying progenitors in the V-SVZ and intermediate progenitor cells in the SGZ). These progenitors will go through a limited round of proliferation before generating neuroblasts, which will migrate to their respective destination (the olfactory bulb for the V-SVZ and the dentate gyrus of the hippocampus for the SGZ) and differentiate into neurons. Despite this extended quiescence, adult NSCs thus actively produce new neurons fated to these CNS regions and supporting fine odor discrimination and odor-reward association (V-SVZ) and learning, memory and spatial pattern separation (SGZ). Several excellent reviews (Bond et al., 2015; Obernier and Alvarez-Buylla, 2019) already exist on adult NSC/progenitors cells, which are not the central focus of this review.

Both the NSCs of the V-SVZ and SGZ are found embedded in the heterogeneous, complex and dense arrangement of cellular and acellular components of their niche (Silva-Vargas et al., 2013; Conover and Todd, 2017). As introduced earlier, common cellular components comprise other NSCs and progenitors, their neuronal and glia progeny, other neurons, different glial subtypes (astrocytes and oligodendrocytes), blood vessels and associated cells (supporting mural cells/pericytes) and resident immune cells (microglia). An additional component of the V-SVZ are ependymal cells lining the surface of the ventricle. Here, we will use the V-SVZ (Figure 1) as a model for the mouse mature niche, with much of this information being relevant to the SGZ as well.

In this niche, multiple tight interactions are spatially arranged between the different cellular components and are supported by a diversity of molecular interactions involving a range of adhesion complexes (reviewed in (Morante-Redolat and Porlan, 2019)). In the V-SVZ, NSCs send a radial basal process to directly contact blood vessels at specific places lacking astrocyte and pericyte coverage (Mirzadeh et al., 2008; Tavazoie et al., 2008) (Figure 1, bubble 1). Parenchymal astrocytes indeed also contact the blood vessels, enwrapping them in a glial sheet involved in blood-brain barrier maintenance and regulation of blood flow throughout the brain ((Mathiisen et al., 2010; Mishra et al., 2016) and reviewed in (Benz and Liebner, 2022)). The mature blood-barrier is indeed nowadays seen as a tripartite structure, with the endothelial cells at its core, and astrocytes and pericytes as supporting yet essential elements ensuring this function (Daneman and Prat, 2015). Astrocytic end-feet of long cytoplasmic extensions contact endothelial cells through the basement membrane, forming rosette-like structures at the surface of CNS blood vessels (Figure 1, bubble 1) (Kacem et al., 1998). In the V-SVZ however, gaps exist in this coverage, allowing some permeability and direct contact with other cells (including the NSCs), thus making the SVZ vasculature apart (Tavazoie et al., 2008). Contact with the vasculature is crucial for NSCs in balancing quiescence and proliferation (Ottone et al., 2014). On the apical side, gap junctions connect NSCs with ependymal cells, which they also attach to via N-cadherin mediated adherens junctions (Figure 1, bubble 2), a key adhesion regulating their quiescent state (Porlan et al., 2014). Meanwhile a single primary cilium per NSC inserts itself within ependymal rosettes to reach the CSF (Figure 1, bubble 2) (Mirzadeh et al., 2008; Shen et al., 2008). NSCs are themselves coupled to each other through gap junctions (Figure 1, bubble 3) (Lacar et al., 2011; Malmersjö et al., 2013). In addition, the neurogenic niche is rich in neurons and innervations coming from further, which form synapses and fill the niche with neurotransmitters. The latter can directly regulate NSC function, as exemplified with GABA maintaining their quiescent state (Song et al., 2012). Finally, microglia extend dynamic processes that can contact other niche populations (Nimmerjahn et al., 2005) and perform multiple roles, from immune surveillance to synaptic pruning, which have been covered by in-depth reviews (Wu et al., 2015; Sirerol-Piquer et al., 2019). This paints a picture of a neurogenic niche abundant in intricate cellular and molecular interactions.

Acellular components also participate in making the niche. First, NSCs are embedded in a basement membrane of extracellular matrix (ECM) (Figure 1, bubble 3). The ECM is composed of a number of core molecules, including collagen(s), elastin, laminin(s), fibronectin, proteoglycans (*e.g.*, perlecan) and tenascin, all present in the adult niche and tightly interacting with NSCs (Kazanis and ffrench-Constant, 2011). In addition, basement membrane-associated fractones (Figure 1, bubble 2) are specialized ECM structures which extend from the blood vessels and can contact NSCs and progenitors (Mercier et al., 2002; Kerever et al., 2007). So far they have been clearly identified only in the V-SVZ, where most of these long and thin, finger-like extensions reach the ependymal surface, establishing direct contact with NSCs and the CSF-filled ventricle (Kerever et al., 2007; Shen et al., 2008). Fractones can also have a bulbar morphology and originate from the ependymal cells themselves, providing anchoring spots and signalling molecules (Nascimento et al., 2018). Finally, interactions between NSCs’ basal processes and blood vessels are mediated by a perivascular basement membrane. This basement membrane is itself made of two layers, the inner vascular layer secreted by endothelial cells and pericytes, and the outer glial layer built by astrocytes, which as such are also critical in providing a structural and signalling belt to blood vessels (Zhao et al., 2015). Therefore, NSCs are in contact with (at least) three different sources of ECM: interstitial, at the vasculature’s attachment and from fractones. Interactions between NSCs and the different ECM layers and structures can be mediated by diverse receptors, with so far most studies on integrins and dystroglycan (reviewed in (Faissner and Reinhard, 2015; Morante-Redolat and Porlan, 2019)). For example, NSCs adhere to blood vessels through the binding of endogenous α6β1 integrin to perivascular laminin, an interaction required for NSCs’ positioning and proliferation (Shen et al., 2008), and fractones can interact with NSCs through α6 integrin (Sato et al., 2019).

Interestingly, choroid plexuses have emerged as an integral component of the adult neurogenic niche (Stolp and Molnár, 2015). They are vascularized epithelial fold-like sacs budding from the ventricular ependymal wall, protruding in the brain ventricles and bathed by the CSF. They also form the blood-CSF barrier. They can be seen as a “distant niche” (Silva-Vargas et al., 2016). Indeed, they are not part of the physical structure embedding the NSCs, and their roles on neuro/gliogenesis go through the CSF, which they produce the bulk of, with the addition of their own, dynamically-regulated secreted factors and of what they pass on from the bloodstream (Ghersi-Egea et al., 2018). As such, considering the angle of this review, the formation of choroid plexuses will be more briefly addressed, yet included to emphasize the development, and possibly coordination, of niche component structures across (distant) space.

### 4.2 Neurogenic niches in the adult zebrafish

One prominent model which has been instrumental in gaining knowledge on NSCs and the process of neuro/gliogenesis in vertebrates is the teleost fish *Danio rerio*, or zebrafish (reviewed in (Barbosa and Ninkovic, 2016; Diotel et al., 2020; Jurisch-Yaksi et al., 2020; Labusch et al., 2020)). In contrast to mammals (and *Drosophila*), the zebrafish displays sustained, extensive and life-long neuro/gliogenesis, supported by NSCs broadly distributed in the CNS and kept from embryogenesis. Many neurogenic zones, and corresponding putative niches, exist in the zebrafish, with varying cellular compositions (Zupanc et al., 2005; Adolf et al., 2006; Grandel et al., 2006; Lindsey et al., 2012). In particular, the zebrafish telencephalon contains regions equivalent to the two main neurogenic niches of adult rodents (V-SZV and SGZ). We will here choose as an example of mature niche the highly-characterized pallium (dorsal region) of the telencephalon (from now referred simply as pallium; Figure 2) (Mueller et al., 2011).

In the adult pallium, NSCs appear as radial glia cells (RGCs), with an apico-basal polarity overtly displayed by polarized cytoplasmic processes, and expressing conserved astroglial markers (Than-Trong and Bally-Cuif, 2015). They also display a single primary cilium peeking in the CSF (Kishimoto et al., 2011) and express components of adherens and tight junction complexes (Figure 2, bubble) (Obermann et al., 2019). They are organised in a continuous epithelial-like sheet, with basal processes contacting blood vessels at the pial surface and with apical processes in the ventricular zone, in contact with the CSF. NSCs are mostly quiescent, a state they exit to maintain population homeostasis (Dray et al., 2021b) and upon specific stimuli, such as regeneration after injury (Alunni and Bally-Cuif, 2016; Zambusi and Ninkovic, 2020). They can follow three different modes of division (Rothenaigner et al., 2011; Than-Trong et al., 2020; Mancini et al., 2023): asymmetric gliogenic, the predominant mode, which generate one RGC and one intermediate (neural, non-gliogenic) progenitor; symmetric gliogenic, which gives birth to two identical RGCs, both having gliogenic fate and typical radial process; and symmetric neurogenic, the least frequent, which generates two intermediate neural progenitors or two neurons. Intermediate neural progenitors appear spatially intermingled with RGCs, which they attach to by tight junctions, and further divide (once to twice) to generate newborn neurons, a process leading to their detachment from the ventricular surface (Mancini et al., 2023). Neurons can also be generated by direct conversion of RGCs, without division or an intermediate progenitor stage (Barbosa et al., 2015; Dray et al., 2021a). Newborn neurons are deposited below the ventricular surface in sequential layers (Furlan et al., 2017), without extensive cell death nor migration happening (with the exception of those produced at the palatial-subpallial boundary and fated for the olfactory bulb, in a process akin to mouse neurons emanating from the V-SVZ (Kishimoto et al., 2011)). The zebrafish CNS does not contain *bona fide* parenchymal astrocytes, except in the optic region (Grupp et al., 2010). Interestingly though, adult pallial RGCs express multiple markers of astrocyte-specific functions (Morizet et al., 2023), which might be fulfilled by the RGCs, a role yet to be demonstrated. A population of oligodendrocytes exist in the pallium (März et al., 2010), and can respond to injury (Baumgart et al., 2012), although their origin remains to be ascertained. Mature ependymal cells are also not widespread in lining ventricular surfaces. Until recently, ependymal-like cells, identified by molecular expression and the presence of beating cilia, were mostly detected in restricted ventricular zones (spinal cord and forebrain diencephalon) (Kishimoto et al., 2011). However, a flurry of recent studies has characterized multi-ciliated cells in multiple CNS regions, including the dorsal and ventral telencephalon (Olstad et al., 2019; Ringers et al., 2020), some formed during development (D’Gama et al., 2021) and some appearing in aged adults (Ogino et al., 2016). These cells are able to move the CSF (Fame et al., 2016; Grimes et al., 2016; Olstad et al., 2019). Yet there are no mature ependymal-like cells in the close structural vicinity of the pallial RGCs. Microglia however are found in the adult pallium (Wu et al., 2020; Pandey et al., 2023), where they can activate in response to injury (März et al., 2011). Their role in homeostatic conditions have been poorly documented.

There is little description of a potential vascular network in the pallium. Blood vessels were detected through electron microscopy deep within the pallial subventricular zone (Lindsey et al., 2012). Blood vessels are also present at the pial surface, contacted by the RGCs (Diotel et al., 2010; 2018) and connected to the host vasculature. It is nonetheless clear that a protective blood-brain barrier exists in the zebrafish (Jeong et al., 2008; Dunton et al., 2021). It is very similar to the mammalian blood-brain barrier, with endothelial walls blocking transcellular permeability through tight junctions, surrounded by a basement membrane, and contacted by perivascular/mural cells (Galanternik et al., 2017). The impact of the vasculature on adult RGCs and neurogenesis is so far unknown.

Finally, zebrafish have choroid plexuses in charge of producing and modulating the CSF (Van Leeuwen et al., 2018; Korzh, 2023), which itself is in direct contact with the apical side of the RGCs. The specific roles of theses choroid plexuses and the CSF on adult neurogenesis also remain to be uncovered.

### 4.3 Neurogenic niches in the *Drosophila* larva

*Drosophila melanogaster* has risen as a powerful system for NSC biology thanks to the conservation of molecular and cellular pathway, its ease of manipulation and the versatility and tractability of its genetics (Homem and Knoblich, 2012). Larval *Drosophila* NSCs (historically called neuroblasts, not to be confused with the mammalian restricted precursors) exist throughout the CNS, in different flavours. Type I NSCS are found both in the central brain and the ventral nerve cord (the equivalent of the vertebrate spinal cord), and divide asymmetrically to produce one intermediate progenitor, the Ganglion Mother Cell (GMC), which will divide once to produce mostly neurons (Truman and Bate, 1988; Pollington et al., 2023). Type II NSCs are found in the central brain as two ventral clusters of eight cells of each side of the midline (Bello et al., 2008; Boone and Doe, 2008; Bowman et al., 2008; Walsh and Doe, 2017). They divide asymmetrically to produce one Intermediate Neural Progenitor (INP), which itself will cycle asymmetrically a few times to give birth to GMCs. As such, Type II lineages are bigger than Type I. In the optic lobe, another CNS region corresponding to the visual processing center, NSCs are produced later, after transitioning from localized neuroepithelia, and follow specific division and proliferation patterns (reviewed in (Nériec and Desplan, 2016; Contreras et al., 2019)). In this review, we will adopt the ventral nerve cord (Type I NSCs only) in late larval stage (Figure 3) as the mature neurogenic niche of *Drosophila*, with however most findings also applicable to the central brain, either for Type II or Type II NSCs. We will explicitly mention when these regions or NSC types diverge in behaviour.

Following a period of quiescence started in late embyrogenesis, larval NSCs cycle actively from mid-larval stage. They are found in a niche made of a diversity of glial cell types (reviewed in (Freeman, 2015; Yildirim et al., 2019; Corty and Coutinho-Budd, 2023)) organised in layers. From the outside, the first niche component is the blood-brain barrier. It is itself composite, made of the neural lamella, a layer of ECM; the perineurial glia, a relatively squamous layer; and the subperineurial glia, which bears the barrier function (Stork et al., 2008; Hindle and Bainton, 2014). The perineurial glia membrane is mechanically linked to the neural lamella through integrin-based focal adhesion complexes, paired with the adhesion molecule Basigin/CD147/EMMPRIN (Figure 3, bubble 1) (Hunter et al., 2020). The subperineurial glia exhibit tight-like junctions, the septate junctions, which provide a physical barrier to paracellular diffusion (Figure 3, bubble 1). In addition, they are equipped with many conserved transporters ensuring chemical filtering (Mayer et al., 2009), while being more permissive for lipid and lipoprotein entry (Brankatschk and Eaton, 2010). As such, the *Drosophila* blood-brain barrier is a neuroprotective barrier, using chemical and physical strategies like in mammals (Juang and Carlson, 1994; Hindle and Bainton, 2014).

Below the subperineurial glia lay the cortex glia. The cortex glia form a striking, dual membrane structure around cycling NSC lineages. It is both a seemingly continuous network infiltrating the whole CNS and encompassing the NSC population; and an individual encasing, called chamber, around each NSC and their whole larval (secondary) lineage (Pereanu et al., 2005; Spéder and Brand, 2018). Mature secondary (larval) neurons and primary (embryonic) neurons display their own individual encasing (shown as a green outline for neurons in Figure 3). Cortex glia have been shown to sustain neurogenesis in multiple fashion, including support of NSC proliferation (Dong et al., 2021), NSC protection from stress (Cheng et al., 2011; Bailey et al., 2015), neuron protection from apoptosis (Coutinho-Budd et al., 2017; Spéder and Brand, 2018; Plazaola-Sasieta et al., 2019) and neuron positioning and features (Plazaola-Sasieta et al., 2019; Banach-Latapy et al., 2023). In addition, secreted cues (netrins and Slit) from the cortex glia bind to their receptors on the NSC membrane, signalling to and regulating the asymmetric division machinery (de Torres-Jurado et al., 2022). Adhesions between cortex glia and NSC membranes also exist, and are at least partially mediated by a Wrapper to Neurexin-IV (homologous to human Caspr/Paranodin) binding (Figure 3, bubble 2) (Banach-Latapy et al., 2023). In addition, several cell adhesion molecules are present within the developing, still immature neuronal lineages contained within a cortex glia chamber, including components of adherens junctions, components of occluding junctions and Neuroglian, the fly homolog of Neurofascin-155 (Figure 3, bubble 3) (Dumstrei et al., 2003; Banach-Latapy et al., 2023). Some sort of physical adhesion seems to also exist between NSCs and their GMC through the midbody generated during asymmetric division (Figure 3, bubble 2) (Loyer and Januschke, 2018).

Below the cortex glia, other types of glial cells are in close association with axonal and synaptic structures. The ensheathing glia, abutting the cortex glia, are found at the interface with the neuropile, the cell body-devoided region where axons meet and synapses forms. While the cortex glia surround the cell bodies and axons of newborn secondary neurons outside of the neuropile, ensheathing glia wrap these axons once they enter this compartment and their function appear important for proper axonal tracts (Spindler et al., 2009). Ensheathing glia also form a diffusion barrier between the cellular and neuropile compartments (Pogodalla et al., 2021). The astrocyte-like glia, with their cell bodies also at the interface between the cortex glia and the neuropile, display a highly-ramified morphology and infiltrate the latter through cytoplasmic extensions with stereotyped tiled locations (Stork et al., 2014; Peco et al., 2016). Astrocyte-like glia are important for axonal pruning and synaptogenesis during pupal metamorphosis (Tasdemir-Yilmaz and Freeman, 2014). So far, there is no evidence that ensheathing and astrocyte-like glia influence neuro/gliogenesis, although we believe they might be revealed to do so, at least through the regulation of newborn neurons. As such, we will not develop them further in this review.

Last but not least, trachea, the branched network of epithelial tubules in charge of supplying oxygen to the tissues, and functioning as a respiratory system, are also present within the CNS. A key question is the importance of oxygen levels in the neurogenic niche. Interestingly, NSCs appear to be more sensitive than neurons, glia or GMCs to oxygen levels, which correlate with the distance to the closest trachea (Baccino-Calace et al., 2020).

### 4.4 State of the field and outstanding questions

So far studies have primarily focused on the signalling side of the niche, investigating the nature and cellular source of the signals modulating NSPC function, both in physiological and stress conditions. While our knowledge has been steadily increasing and has uncovered a tremendous diversity of cues and impacts on NSCs, much is still left to do to map the whole signalome of the niche, how it changes under a range of stress/pathological conditions, and the interplay between the different signals.

Yet, the architectural aspect of the NSC niche is even less understood and still very poorly explored. Many central questions are left to be investigated.

1. What is the developmental origin of the adult niche: contribution of components; plasticity?
2. How does the niche architecture form: timescale and dynamics; cell types involved; cellular and molecular mechanisms?
3. What are the different scales of the niche: local, regional, population-wide? What are the associated topological rules?
4. Which roles does niche architecture perform on NSC and niche functions? What is the relationship with the different scales?

## 5 Building a neurogenic niche

All in all, the (local) neurogenic niche is a dense microenvironment, rich in a diversity of cellular and molecular interactions, and formed by components of different origins yet brought together in one place. We will now describe the developmental events building the architecture of the mature niche from the start of neuro/gliogenesis, when “a” niche cannot actually be properly localised. We will also discuss some functions of these niches when they appear tied to architectural features.

### 5.1 The developing mouse neocortex

The adult V-SVZ niche originates from a highly proliferative neuroepithelium lining the brain’s ventricular system during development, but which becomes significantly limited later, localized along the lateral wall of the lateral ventricles. As such it can be regarded as a spatially restricted continuation of the embryonic V-SVZ. We will thus focus on the V-SVZ of the developing neocortex (in the dorsal telencephalon) to convey the main principles behind the formation of a neurogenic niche (Figure 1). Of note, an outstanding review from (Bjornsson et al., 2015) has described, and bridged, the developing and adult niche in the mouse (composition and signalling).

#### 5.1.1 Neural progenitors

The first step of developmental neuro/gliogenesis is neuroectodermal specification (or neural induction (Lumsden and Krumlauf, 1996)) between embryonic day (E)6.0 and E8.5, leading to the formation of a neuroepithelial sheet. Developmental cortical neuro/gliogenesis (reviewed in (Paridaen and Huttner, 2014; Villalba et al., 2021) starts around embryonic day E9.5, with the neuroepithelial cells as primary neural progenitors. Neuroepithelial cells create a tube structure with a central canal and exhibit characteristics of epithelial cells, forming lateral connections through adherens and tight junctions and displaying apicobasal polarity (Figure 1, bubble 4). They are organised as a pseudo-stratified neuroepithelium, a result of interkinetic nuclear migration (Noctor et al., 2001). After several initial rounds of division to amplify their pool, neuroepithelial cells transform into radial glia cells (RGCs) around E10.5. RGCs’ cell bodies are still mechanically linked to each other by adherens junctions (but no more tight junctions) at the apical side, an interaction critical to their behaviour and to the integrity of the ventricular zone (Rašin et al., 2007). RGCs are bipolar, displaying both apical and basal processes (Noctor et al., 2002). Apical processes, harbouring a primary cilium, peek into the ventricle, filled with the CSF. Primary cilia are immotile microtubule-based organelles, present in most cells, and which serve as a signalling plateform, hence gaining the nickname of “cell’ antenna”. They are abundantly present in the mammalian developing CNS, in most of the niche cell types, and as such their alteration has been associated with many neurodevelopmental disorders (Liu et al., 2021). In embryonic RGCs, the primary cilium is disassembled then re-assembled during each cell division, in a tight relationship with centriolar inheritance between the daughter cells. Primary cilium’s ablation in RGCs disrupts mitotic axis and the cellular outcome of division (Foerster et al., 2017), while disruption of some of its signalling roles alters apico-basal polarity (Higginbotham et al., 2013). The elongated basal processes of RGCs extend towards the outer (pial) surface of the brain, attaching to the basement membrane of pial vasculature or to the pial membrane itself (Figure 1, bubble 5). RGCs undergo both symmetric and asymmetric divisions. Self-renewal through asymmetric division can either generate a neuron, a glial cell or an intermediate progenitor, which itself will divide further to produce neurons. Of note, in the developing neocortex, two other (and smaller in the mouse) populations of NSPCs exist: the short neural precursor cells, which lack basal attachment and exclusively produce neurons through differentiative divisions; and the outer/basal RGCs which lack apical attachment and generate basal intermediate progenitors (reviewed in (Agirman et al., 2017; Kalebic and Huttner, 2020)). Basal RGCs are proposed to be a key support for cortical expansion during evolution.

Gliogenesis tends to happen later than neurogenesis at the level of the population (reviewed in (Clavreul et al., 2022)). In particular, in early postnatal development, progenitor cells primarily generate glial cells. The remaining RGCs mostly differentiate into ependymal cells, or into various types of glial cells, such as astrocytes. A restricted number of RGCs still persist (Fuentealba et al., 2015; Berg et al., 2019), remaining in localised microenvironments which will become the adult neurogenic niches. The basis of these adult niches is thus made by the diverse components assembled during development, and which will be remodelled later on.

#### 5.1.2 Extracellular matrix

One niche component in direct contact with NSPCs is the ECM, from the basement membrane or from fractones. The ECM is composed of a number of core molecules and can also sequester diverse growth factors and cytokines. All these can work as signalling molecules (ligands of cellular receptors) *per se*, and together also provide a mechanical scaffold to the cells. In the embryonic CNS, the core ECM components all appear expressed (reviewed in (Kazanis and ffrench-Constant, 2011)), as well as many of their cellular receptors (integrins; syndecans; dystroglycan) (Lathia et al., 2007). The cellular sources of the different ECM components have not been precisely characterized, except for the perivascular basement membrane (see further) and the expectation that some should come from the cells they surround. Whether the composition of the ECM varies during developmental neuro/gliogenesis, and between the developing and adult neurogenic niches, remain poorly understood.

The study of ECM function in the developing CNS has uncovered both signalling and mechanical roles, roles often difficult to disentangle. Several studies have assessed the effect of removing the integrin subunits serving as laminin’s receptor in NSPCs, whose apical and basal processes are both anchored to basement membranes via integrin complexes (Figure 1, bubbles 3 and 5). These removals all resulted in the disruption of basal process attachment, yet with different outputs on NSPC function, leading to either death (Radakovits et al., 2009) and alteration of proliferation axis (Loulier et al., 2009) for integrin ß1, or to no detectable impact for integrin α6 (Haubst et al., 2006). Altered expression of the heparan sulfate proteoglycan Perlecan both led to structural disruption of the neuroepithelium by disorganizing the ventricular basement membrane (Costell et al., 1999), and to decreased NSPC proliferation through its control on Sonic Hedgehog signalling (Girós et al., 2007). The knockout during embryonic development of another ECM component, Tenascin C, also affects NSPC proliferation (Garcion et al., 2004). Fractones represent another form of ECM structures which is already present early on during CNS development, at the neuroepithelium stage in the V-SVZ (Mercier, 2016). Little is known yet on their roles at this stage and how they relate to adult fractones.

In addition to ECM production, several cell types start to populate the developing neocortex, progressively assembling a cellular niche structure.

#### 5.1.3 Neurons

Newborn neurons migrate away towards the pial surface (/cortical plate), where they fully differentiate and establish connections. Neurons generated directly from RGCs must initially detach from the ventricular surface by breaking N-cadherin-based adherens junctions (Rousso et al., 2012). In contrast, neurons born by indirect neurogenesis from intermediate progenitors, which have themselves already detached and migrated basally, do not require this detachment step. Newborn neurons then use RGCs’ basal processes as a guiding scaffold (Figure 1, bubble 6), which they attach to through gap junction hemichannels (Elias et al., 2007) and/or by relying on a dynamic, Rab GTPases-regulated binding to N-cadherin (Kawauchi et al., 2010; Shikanai et al., 2011). These neurons, arranged in consecutive subtype-specific layers depending on their time of birth (early-born in deep layer and late-born in superficial layer) can in turn regulate the behaviour and functions of NSPCs through paracrine mechanisms (Xing and Huttner, 2020).

#### 5.1.4 Vasculature and the blood-brain barrier

A key component of the mouse neurogenic niche is a complex network of blood vessels which develop and grow alongside its emergence and expansion (for extensive reviews on neurodevelopmental angiogenesis, see (Saunders et al., 2012; Paredes et al., 2018; Biswas et al., 2020; Peguera et al., 2021; Vogenstahl et al., 2022)). Angioblasts, the vascular progenitors of the endothelial cells, are recruited to the outside of the neural tube, where they form the perineural vascular plexus (see top vessel on Figure 1) around E8.5-E9.5. They then initiate angiogenesis at around E9.5-E10, at the time neuroepithelial cells convert into RGCs. Vasculogenesis and neuro/gliogenesis thus appear temporally coupled. Endothelial tip cells lead vascular sprouts from the perineural vascular plexus, breaking through the pial basement membrane. These primitive vessels then infiltrate the CNS tissue and, guided by NSPC-derived VEGF, quickly extend radially towards the ventricle. There, they form branches which connect with neighbouring ones to create the intraneural vascular plexus (Engelhardt and Liebner, 2014). The intraneural vascular plexus further extends, also developing additional capillary meshes reinforcing this early vascular framework, and becomes the periventricular vascular plexus. The periventricular vascular plexus basically corresponds to the dense, grid-like vascular network spanning the neurogenic niche, connected to the perineural vascular plexus. This entire process occurs rapidly (Ziegler et al., 2014), and is vital for delivering oxygen as the brain expands.

The formation of the blood-brain barrier happens concomitantly to angiogenesis and vascularization of the CNS, with endothelial cells setting up tight junctions and expressing specialized transporters. A primitive blood-brain barrier is already present at E15, then matures and is stabilized by the addition of cellular components (Daneman et al., 2010; Ben-Zvi et al., 2014). Pericytes in particular are recruited to forming blood vessels during vascularization of the developing CNS, where they are critical for the formation and maintenance of the blood-brain barrier (Daneman et al., 2010). While pericytes are also associated with the vasculature in other tissues, they are more abundant along the neurovasculature than in any other part of the body. These contractile mural cells (Armulik et al., 2011), of heterogeneous origin including the neural crest (Dias Moura Prazeres et al., 2017), wrap around endothelial cells through long cytoplasmic processes.

Blood vessels and NSPCs mutually influence each other’s development and architecture. First, correct vasculature growth and topography are crucial for defining the neurogenic niches during CNS development. NSPCs are indeed preferentially found in the close vicinity of blood vessels, in particular close to tip cells during vasculature branching, which contact them through filopodial extensions (Javaherian and Kriegstein, 2009; Di Marco et al., 2020). Disturbing blood vessel growth in the embryonic brain or in embryonic brain cultures transfers NSPCs to non-neurogenic regions (Javaherian and Kriegstein, 2009; Li et al., 2013), a striking observation suggesting that the vasculature directs niche location. In a contrasting picture, neurogenic niches tend to be hypoxic, a feature supporting RGC proliferation (Mohyeldin et al., 2010). Blood-dependent oxygen levels in the developing CNS indeed seem to participate in balancing proliferation and differentiation of NSPCs, with hypoxia favouring the former while oxygenation promotes the latter in a HIF1a-dependent manner (Lange et al., 2016). Even more, progenitor identities can depend on oxygen level (Wagenführ et al., 2015). The timing and pattern of vascularization of the developing CNS is thus a driver of niche morphogenesis.

While the influence of the vascular niche on neuro/gliogenesis depends on its role of an interface with blood-borne factors, it is also a structural scaffold, bearing direct physical interaction. It is indeed able to regulate RGC proliferation through direct cell-cell contacts. Apical end-feet of RGCs anchor to blood vessels of the periventricular plexus through integrin-based adhesions (Figure 1, bubble 5), an interaction regulating RGC proliferation (Tan et al., 2016). Of note, different subtypes of progenitors in different CNS regions are more or less dependent on this association, showing that not all niches are equal. Direct contact of RGCs by endothelial tip cell filopodia can also modulate RGC proliferation by extending their mitotic phase, what ultimately triggers earlier neural differentiation while limiting pool amplification (Di Marco et al., 2020). In addition, RGCs’ attachment to pia vessels via their basal processes and integrin/ECM interactions influences the positioning of newborn neurons (Figure 1, bubble 5) (Graus-Porta et al., 2001; Haubst et al., 2006; Radakovits et al., 2009; Segarra et al., 2018).

Reciprocally, NSPCs and progeny are important for the extent and patterning of CNS vascularization. First, the key angiogenic factor VEGF is expressed by NSPCs (Hogan et al., 2004; James et al., 2009) and neurons (Himmels et al., 2017), and is essential for the different steps of CNS vascularization, including the formation of the perineural vascular plexus and the ingression of blood vessels into the periventricular zone. Earlier on, neuroepithelial cells also secrete Wnt7a/b, activating the corresponding pathway in endothelial cells, what in turn ensures proper angiogenesis and blood-brain barrier function (Daneman et al., 2009). A delicate balance in Wnt signalling in endothelial cells, controlled by the NSPCs, is actually important for the proper growth and stabilization of periventricular blood vessels (Stenman et al., 2008; Ma et al., 2013). Astrocytes also play a key role in regulating developmental angiogenesis, both for the vascularization process (Ma et al., 2012) but also, more prominently, for blood-brain barrier function, through their direct interaction with endothelial cells’ basement membrane.

#### 5.1.5 Glia: astrocytes and oligodendrocytes

Astrocytes indeed are another pivotal component of the neurogenic niche. They appear relatively late in the process, following gliogenesis (reviewed in (Clavreul et al., 2022)). Around E16.5, RGCs switch to generate astrocytes and oligodendrocytes at the expense of neuronal lineages, producing glial rather than neural (intermediate) progenitors. This switch is seen at the population level, while RGCs’ potential is divided into neurogenic-only, gliogenic-only or bipotent (nicely synthesized in (Kriegstein and Alvarez-Buylla, 2009)). RGCs can also directly convert into astrocytes perinatally. Astrocytes are born immature and migrate to their final position before acquiring markers of fully mature astrocytes, a process occurring up to perinatal development. Astrocytes are a heterogeneous cell population, with gene expression, functional and spatial differences (Molofsky and Deneen, 2015). In addition to their role in supporting neuronal functions, which have been covered before in (Durkee and Araque, 2019; Bonvento and Bolaños, 2021), they are in tight relationship with the developing vasculature. Not only do they participate in angiogenesis (Ma et al., 2012) and express several angiogenic factors, they are also an integral part of the blood-brain barrier.

#### 5.1.6 Microglia

In contrast, microglia are an early component of the neurogenic niche and are recruited from the outside. Microglia are tissue-resident macrophages, born during development in the yolk sac and derived mostly from erythro myeloid progenitors, with a potential contribution from primitive macrophage (Alliot et al., 1999; Ginhoux et al., 2010; Schulz et al., 2012; Perdiguero et al., 2014; Ferrero et al., 2018). In the mouse, erythro myeloid progenitors form in the yolk sac at E8.5 and microglia precursors then migrate to their tissue of residence, entering the CNS around E9.5, when angiogenesis starts. Primitive blood vessels and meninges have been proposed to serve as entry points (Navascués et al., 2000; Ginhoux et al., 2010), and neuronal apoptosis as one attracting cue. There microglia follow a stepwise developmental program (early microliga > pre-microglia > adult microglia) allowing them to adapt to their novel environment, and leading to their adult form (Matcovitch-Natan et al., 2016). They first proliferate until the post-natal period and colonize the entire CNS, while showing preferential, dynamic localizations, with transient hotspots on regulated migratory paths (Swinnen et al., 2013). Some of these paths might be influenced by RGCs, which directly interact with microglia during early development (Rigato et al., 2011; Rosin et al., 2021) and also appear to be important for their recruitment (Arnò et al., 2014). They might also use the expanding periventricular vasculature as a migratory track, with half of the microglia found closely associated with blood vessels at E14 (Hattori et al., 2022). In a reciprocal fashion, microglia appear to support angiogenesis (Fantin et al., 2010).

Microglia are very diverse in shapes and functions (from amoeboid-like to highly ramified), one linked to the other, and are also dependent on their state of maturation and to their location within the CNS. During development, microglia have been involved in regulating NSC proliferation and differentiation. While microglia are difficult to specifically deplete, several studies using complementary genetic or chemical ablations have been able to assess how their loss of function impacts CNS development. Conditional depletions of microglia in the mouse developing neocortex showed that microglia support stem cell differentiation into intermediate progenitors (Arnò et al., 2014; Hattori and Miyata, 2018). Chromatin-dependent regulation of microglial features (shape, metabolism) is also important for correct NSPC self-renewal and differentiation during embryonic development, through modulation of the Wnt/β-catenin signalling pathway (Su et al., 2022). In the rat developing brain, microglia activation in the ventricular zone promotes neuro/gliogenesis (Shigemoto-Mogami et al., 2014). Microglia also help building and refining neuronal circuits, using signalling as well as their pruning and phagocytic abilities. In particular, an excessive number of neurons are generated during early brain development, and many of them undergo programmed cell death before birth (Blaschke et al., 1998; Yeo and Gautier, 2004). Microglia are found to concentrate in zone of neuronal death in various CNS regions and respond to death signals by engulfing dying neurons with their processes (Ferrer et al., 1990; Wakselman et al., 2008; Rigato et al., 2011). Microglia can also establish cell contacts with neuronal cell bodies to shape their wiring (Squarzoni et al., 2014).

#### 5.1.7 Ependymal cells

Ependymal cells are a very late player in niche morphogenesis, and present in the SVZ in contrast to the non-ventricular SGZ. They are multiciliated epithelial cells which line all brain ventricles, forming a mostly continuous cellular barrier with the CSF. The coordinated and oriented beating of their cilia moves this fluid through the ventricles. Ependymal cells are post-mitotic and come from the differentiation of RGCs at late developmental stages (E14-16), arising from terminal symmetric divisions (Spassky et al., 2005; Ortiz-Álvarez et al., 2019; Redmond et al., 2019). They further need to mature post-birth to adopt their adult profile (Del Bigio, 2010). Ependymal cells are linked to each other by adherens junctions, but do not display tight junctions except at few restricted locations. In addition, they assemble in rosette structures around grouped apical processed of several RGCs, similar to the pinwheel structures of the adult neurogenic niche (Figure 1, bubble 2) (Mirzadeh et al., 2008; Codega et al., 2014). These pinwheel structures provide restricted breaks in the ependymal barrier, offering exchange points with the CSF. Different subtypes of ependymal cells exist (tanycytes, radial and cuboidal), classified based on their morphology, location and molecular markers, and potentially fulfilling different functions. The role of ependymal cells in the developing niche is not characterized, and possibly limited due to their late arrival. In the adult niche, they are essential in regulating neuro/gliogenesis, both as a source of signalling molecules which influence the behaviour and fate of nearby NSCs, and indirectly as a main controller of the CSF (reviewed in (Quaresima et al., 2022)).

#### 5.1.8 Choroid plexus: an example of a distant niche

There are four choroid plexuses, one in each brain ventricle (two lateral, third and fourth). While they diverge in morphology and function, they share a similar cellular structure, made by two major cell types. The first element is a monolayer of cuboidal epithelial cells of neuroectodermal origin and in continuity with the ependymal cells of the ventricular wall. They are also ciliated, but, in contrast to the latter, they are connected by tight junctions, providing a barrier to paracellular diffusion, and exhibit microvilli structures to increase their total surface. The second element is a network of fenestrated capillary blood vessels connected to the brain circulation system. Pericytes and immune cells are found in the space between capillaries and epithelial layer.

The formation of the different choroid plexuses is a complex process bringing different cell types (epithelium and blood vessels), from different origins (neuroectoderm and mesoderm respectively) together. Presumptive choroid plexuses’ territories in the neuroepithelium can be identified from as early as E.8.5 (Thomas and Dziadek, 1993; Awatramanil et al., 2003), specified through the coordinated action of signalling patways (Notch, BMP) to repress neural fate. Choroid plexuses’ epithelia then enter a maturation and differentiation stage, with their structures becoming apparent around E11.5 following morphological changes (Sturrock, 1979). In particular, the epithelium undergoes thinning, transitioning from pseudostratified to columnar and eventually cuboidal, forming a proper single layer as it starts to extrude into the ventricles (Dziegielewska et al., 2001). Mature choroid plexus epithelial cells are mostly post-mitotic, and as such growth during development occurs by incorporating cells from the proliferative zone at its base. During these morphogenetic changes, the underlying, developing vascular network in the mesenchyme ends up being enveloped by the growing epithelium. This is not only a result of physical topological constraints, as the development of the two components of the choroid plexuses appears coordinated: signalling from the neuroepithelium is proposed to trigger vascular differentiation (Broom et al., 2012), and growth (Nielsen and Dymecki, 2010).

While still maturing and growing, choroid plexuses already influence neuro/gliogenesis during development by modulating CSF composition (reviewed in (Zappaterra and Lehtinen, 2012; Johansson, 2014)). The early CSF indeed carries, in a dynamic fashion, a range of signalling molecules, including, and not restricted to, growth factors, BMP ligands, Wnt ligands, and retinoic acid. All these factors have the potential to regulate NSPC function and neuro/gliogenesis. As examples, CSF-borne Sonic Hedgehog supports RGC division in the cerebellar ventricular zone (Huang et al., 2010), and in the developing cortex Insulin-like growth factor 2 binds to its receptor on the apical process of the RGCs, stimulating their proliferation (Lehtinen et al., 2011).

### 5.2 The zebrafish pallium

The zebrafish pallium displays continuous neurogenesis, and a neurogenic niche, which can be observed and studied throughout life, simplifying the link between developmental and adult phases. Well-described components of the neurogenic niche in the zebrafish pallium are the RGCs themselves and their neuronal progeny. Other identified, but poorly characterized, components are an oligodendrocyte population, the microglia and some vascular system. In this light, we propose here to mostly focus on the formation of the neurogenic cells (RGCs) during development (embryonic (0–48 h post-fertilization, hpf) and larval (3–90 days post-fertilization, dpf) stages, with hatching (day 2-3 post-fertilization) in between) (Figure 2).

#### 5.2.1 Neural progenitors

Following neuroectodermal specification, the neuroepithelium, which will shortly progress into RGCs, experiences a number of morphogenetic events (formation of a neural keel then rod) to ultimately generate a neural tube (around 18–20 hpf (Araya et al., 2016)), with a typical central canal filled with CSF. However, this canal structure is transient, and the neuropithelium in the telencephalon undergoes an eversion process, resulting in the exposure of the proliferative zones on the outer surface of the telencephalon (Nieuwenhuys, 2011). As such, and in contrast to mammals, the CSF is not encased in a canal but flows at the surface of the brain, lined on the other side by the *tela choroidea*, an epithelial-like sheet formed during eversion. Zebrafish telencephalic eversion is a complex multi-step morphogenetic process, starting shortly before 2 dpf (18 hpf) and completed at 5 dpf, and thus covering the beginning of neurogenesis during embryogenesis (Folgueira et al., 2012). Neuroepithelial cells, which are first organised as a pseudo-stratified epithelium (Hiscock et al., 2018), indeed convert into proper RGCs, with a radial, apico-basal polarity around mi-embryogenesis (22 hpf) (Dong et al., 2012). They are at least morphologically detectable at this stage, with some molecular markers expressed earlier. At this early stage, RGCs already perform a majority of asymmetric cell divisions along with some symmetric divisions (generating either two RGCs or two restricted neural progenitors) or differentiation into neurons. Interestingly, the daughters issued from asymmetric division adopt different positions along the apico-basal axis of the RGCs (with the basal daughter keeping the self-renewal ability), highlighting some degree of relationship between topography and fate. Ultimately, differential Notch-driven signalling between the daughter cells will decide of their fate (Dong et al., 2012). Some progenitors (Oligo2^+^), appearing from 30 hpf, give birth to oligodendrocytes, which start maturing at 48 hpf, and fully myelinate around 72 hpf (Ackerman and Monk, 2016). From this time, RGCs will keep proliferating to sustain the continuous growth of the zebrafish pallium, albeit with a shift towards a mostly quiescent state in adult (Dirian et al., 2014; Furlan et al., 2017).

#### 5.2.2 Microglia

Embryonic zebrafish microglia derive from the yolk sac (Herbomel et al., 1999), like in mammals, yet with a unique origin from primitive macrophage (Ferrero et al., 2018). They start to spread as microglial precursors from around mid-embryogenesis (22 hpf) and slowly start to colonize the brain (including the telencephalon) from 35 hpf by crossing cellular barriers rather than using the blood circulation (Herbomel et al., 2001). There, they undergo a phenotypic shift, from an ameboid-like shape to a ramified morphology. Of note, a second wave of microglial colonization, derived from the hematopoietic stem cells, happens later at 14 dpf and participates to the adult pool (Xu et al., 2015; Ferrero et al., 2021).

In a variety of locations in the developing zebrafish brain, microglia have been shown to phagocytose dying neurons (Peri and Nüsslein-Volhard, 2008), which recruit them through the release of lysophosphatidylcholine and its binding to its microglial receptor (Casano et al., 2016; Xu et al., 2016). Cytokine signalling also seems to be an attractant, in conjunction or not with apoptotic cues (Herbomel et al., 1999; Wu et al., 2018). Beyond the regulation of neuronal number through their phagocytic role, no studies have so far uncovered a role for microglia in developmental neurogenesis.

#### 5.2.3 Vasculature and the blood-brain barrier

To our knowledge, there is no description of vasculogenesis and angiogenesis specifically in the developing telencephalon. We will briefly describe the general process of brain vascularization in other regions (reviewed in (Gore et al., 2012; Quiñonez-Silvero et al., 2020)), and its interactions with neurogenesis. After angioblast specification and their migration to the midline, vasculogenesis starts and gives rise to the perineural vascular plexuses (Hogan et al., 2004) surrounding the brain and spinal cord, around 20 hpf. Angiogenic endothelial tips sprout from these plexuses and invade the brain (30–32 hpf for midbrain and hindbrain). There, they will go into rounds of angiogenesis, branching and connecting to other vessels to form a dense intraneural network inside the brain (Fujita et al., 2011; Ulrich et al., 2011). These events are highly dependent on VEGF signalling. The establishment of the blood-brain barrier function is temporally coupled to angiogenesis, with CNS protected from 2.5-3 dpf (Quiñonez-Silvero et al., 2020). Pericytes are also present in the zebrafish, and support blood-brain barrier establishment and function (Galanternik et al., 2017; Stratman et al., 2017; Bahrami and Childs, 2018). The whole process is thus very similar to the sequence of events in the mouse. Using a mutant (*cloche*) in which most of the vasculature is absent, studies have found that neurogenesis and/or formation of neuronal networks was impaired in some regions (cerebellum and optic tectum) but not others (hindbrain) (Ulrich et al., 2011; Taberner et al., 2020). In a reciprocal fashion, RGCs’ activity seems important for brain vascularization. Developing blood vessels appear closely associated with RGCs, whose presence is required for their growth (Umans et al., 2021). Of note, in the developing spinal cord, RGCs regulate the extent of sprouting from venous trunk vessels, in a VEGF-dependent manner (Matsuoka et al., 2016).

#### 5.2.4 Tela choroidea

In the telencephalic region, the development of a choroid plexus-like tissue start with the formation of the *tela choroidea* during eversion (Folgueira et al., 2012; Turner et al., 2022). Around 18 to 22 hpf, an out-pocketing of the ventricular surface between the telencephalon and diencephalon creates a fold with a diamond-shaped roof of neuroepithelial origin. Between 2 to 5 dpf, accompanying pallial growth, this roof expands forward over the pallium to cover it, forming the wide and thin *tela choroidea*. *Tela choroidea* cells might become ciliated from around 14 dpf (D’Gama et al., 2021). A folded and more conventionally structured choroid plexus is found posterior to the telencephalon. There is still limited documentation regarding its formation and function during zebrafish development (Bill et al., 2008; García-Lecea et al., 2008; Henson et al., 2014; Van Leeuwen et al., 2018).

### 5.3 The *Drosophila* ventral nerve cord

The mature *Drosophila* neurogenic niche in larval stages is articulated around NSC lineages and their neuronal progeny, with the blood-brain barrier and the cortex glia as main known structures and regulators. We will follow the formation of this niche around Type I NSCs from early embryo to late larva, in spatial and temporal continuity within the ventral nerve cord. As mentioned earlier, most findings also apply to central brain Type I and Type II NSCs.

#### 5.3.1 Neural stem cells and neurons

*Drosophila* Type I NSCs appear at embryonic stages following delamination from the neuroectoderm (Campos-Ortega, 1993; Bossing et al., 1996). They proliferate extensively through rounds of asymmetric division, producing embryogenesis-born neurons (called primary neurons) as well as glial cells, which will form the larval CNS at hatching. NSCs come in various types, producing both neurons and glia (“neuroglioblast”), only neurons (“neuroblast”) or only glia (“glioblast”) (Udolph et al., 1993; Bossing et al., 1996; Bernardoni et al., 1997; Schmidt et al., 1997; Schmid et al., 1999; Altenhein et al., 2015), hinting that some fate restriction might already be in place early. At the end of embryogenesis, they slow down their proliferation, shrink in size (3–5 μm), and ultimately enter a quiescent stage, characterized by their mitotic silence and the existence of a primary actin-rich and microtubule-rich (Li et al., 2017) membrane extension which reaches the neuropile (Truman and Bate, 1988; Chell and Brand, 2010). They stay quiescent until early larval stage, when they reactivate and start proliferating again (Prokop and Technau, 1991; Ding et al., 2020). Larval feeding triggers their reactivation. More precisely, circulating amino acids are sensed by the fat body (the invertebrate equivalent on the liver and adipose tissue), which sends a yet-to-be identified signal to the CNS (Britton and Edgar, 1998). As such, the fat body behaves as a “distant” niche for fly NSCs. In turn, insulin signalling is activated in NSCs, initiating the whole reactivation process (Chell and Brand, 2010; Sousa-Nunes et al., 2011). Reactivation encompasses their growth back to their original size (10–12 μm) and their re-entry into mitosis. They also lose their membrane extension, which appears retained only for the first division (Gujar et al., 2023). Then, NSCs will actively divide in an asymmetric fashion to produce secondary neuronal lineages, which will mature later on to form the majority (90%) of the adult CNS (White and Kankel, 1978; Truman and Bate, 1988). NSCs then appear to differentiate or die (Bello et al., 2003; Maurange et al., 2008) during late larval/early pupal stage. Of note, neuro/gliogenesis still happens in the adult fly, primarily within the visual system, with the identity, and link to developmental stages, of dividing cells yet to be ascertained (Kato et al., 2009; von Trotha et al., 2009; Fernández-Hernández et al., 2013; Simões et al., 2022). Both during primary and secondary neurogenesis, Type I and Type II NCS undergo a series of temporal transitions which, in combination with spatial identity, will generate neuronal diversity (reviewed in (Doe, 2017)). Most of the NSCs generated during embryogenesis persist during larval stage, with the exception of an apoptotic wave in the abdominal part of the ventral nerve cord (Maurange et al., 2008).

NSCs are relatively regularly spaced within the central brain or ventral nerve cord. No specific adhesion or interaction has been found so far between NSCs, in embryonic or larval stage, in accordance at least with their separation by the cortex glia during the latter. Very little is also known regarding neurons’ role as a niche for NSCs. Primary neurons are scattered in between NSCs, and individually encapsulated by the cortex glia membrane (Coutinho-Budd et al., 2017). Secondary neurons from the same lineage stay together with their mother NSC within a cortex glia chamber until they mature (Pereanu et al., 2005). This clustering happens, at least in part, through a mechanism of differential adhesion using complexes containing immunoglobulin-like domains (Banach-Latapy et al., 2023). It involves intra-lineage homophilic interactions by Neuroglian, and a weaker interaction between Neurexin-IV in the NSC lineages and Wrapper in the cortex glia (Figure 3, bubble 3). While these adhesions appear to impact axonal features of the secondary neurons, no apparent phenotype is detected at the level of the NSCs themselves. Components of the septate junctions also appear enriched in developing NSC lineages with no further role identified so far. Similarily, adherens junctions are detected between cells from the same NSC lineage. Conflicting results exist on the importance of these adherens junctions. One study using a dominant negative form for Shotgun, the fly E-cadherin, observed strong alterations of the cortex glia structure when clonally expressed in the CNS, including in the cortex glia (Dumstrei et al., 2003). In contrast, another study found no effect when using cell type-specific RNAi knockdown or null mutant clones in the NSC lineages (Banach-Latapy et al., 2023). All in all, still little is known about the complexity of physical interactions between newborn neurons, and their potential impact on neurogenesis.

#### 5.3.2 The blood-brain barrier

The blood-brain barrier includes the neural lamella, the perineurial glia and the subperineurial glia.

Little is known on the ECM of the neural lamella, either the source(s) of its components and its functions towards NCSs and neurogenesis. The ECM sheath is present from embryonic stage 16 (13–16 h after egg laying (AEL)) (Stork et al., 2008) and remodels to accommodate CNS growth during development, a remodelling depending on the expression of matrix metalloproteases in the underlying perineurial glia layer (Meyer et al., 2014). The heparan sulfate proteoglycan Perlecan, a core ECM component, appears important for NSC reactivation and for the overall shape of the CNS (Datta, 1995; Voigt et al., 2002; Park et al., 2003). Collagen-IV (referred to as Viking in *Drosophila*) in the neural lamella appears to have, at least in part, an external origin, being produced and secreted by the fat body (Pastor-Pareja and Xu, 2011). How the different components are assembled, and their dynamics along the development of the CNS at larval stages, are unknown.

Perineurial glia cells, in direct contact with the neural lamella, are generated during late embryogenesis and must migrate to the CNS surface, although to our knowledge their exact origin and the migratory process have not be characterized. They do not touch each other at the beginning of larval stages, but do so later one, following rounds of proliferation (Ito et al., 1995; Awasaki et al., 2008; Stork et al., 2008). At late larval stage, when NSCs proliferate, they form a complete monolayer sandwiched between the neural lamella and the subperineurial glia. Perineurial glia are understudied, and little is known on their function and interactions with other components of the niche. One study showed that perineurial glia participate in NSC reactivation through the expression and function of the heparan sulfate proteoglycan Dally-like (Kanai et al., 2018). Reciprocally, perineurial glia depend on BMP signalling from NSCs for their survival. Curiously, the same study shows that direct contact seems to be possible between NSCs and perineurial glia at reactivation time, during early larval stage, despite an interleaved subperineurial glia layer.

Subperineurial glia cells themselves are generated during embryogenesis from specific NSCs (amongst which NBs 1-1A, 2-2T and 5–6A) (Beckervordersandforth et al., 2008). They migrate to the surface of the CNS and grow laterally until they touch each other and form occluding/septate junctions at the point of contact (Schwabe et al., 2017). The subperineurial glia seal and provide a barrier to paracellular diffusion by late embryonic stages (stage 17, from 15 h after egg laying) (Bainton et al., 2005; Schwabe et al., 2005). During larval stage, to accommodate the expanding volume of the CNS driven by NSC proliferation, subperineurial glia grow through endoreplicative mechanisms (mainly endomitosis), without cell division, a strategy crucial for the maintenance of its barrier function (Unhavaithaya and Orr-Weaver, 2012). Rather, they remodel their septate junctions through stretching to keep up with their growing surface (Babatz et al., 2018). Part of their growth seem to be fueled by the nutrition-dependent activation of the PI3K/Akt at early stages, during CG expansion (Yuan et al., 2020). The subperineurial glia is also a sensing interface of this nutritional signal, mediating its impact on NSC reactivation (Chell and Brand, 2010; Spéder and Brand, 2014). Such sensing triggers the production and release of *Drosophila* Insulin-like peptides (dIlps), a process dependent on gap junction coupling between subperineurial glial cells (Figure 3, bubble 4). More precisely, nutritional cues trigger calcium oscillations which are synchronized across the entire subperineurial layer through gap junction channels. This is proposed to result in an en masse regulated secretion of dIlps, required for synchronous NSC reactivation. dIlps further bind to the insulin receptor at the surface of the NSCs, resulting in the activation of the conserved PI3k/Akt pathway (Chell and Brand, 2010; Sousa-Nunes et al., 2011). PI3k/Akt signalling in the NSC is necessary and sufficient for reactivation. Interestingly the assembly of heteromeric gap junctions (made by the innexins 1 and 2) is lost after reactivation (Spéder and Brand, 2014), suggesting a coordination between subperineurial glia architecture and metabolic cues. So far, study of the subperineurial glia morphogenesis has mainly focused on intrinsic mechanisms. There is no well-characterized physical association between perineurial and subperineurial glia, nor between the subperineurial and the underlying cortex glia in the CNS, except for one documented instance of gap and adherens junctions in the latter case of interaction (Pereanu et al., 2005). How these cells talk to each other, at the signalling and adhesion levels, remain to be uncovered.

#### 5.3.3 Cortex glia

Cortex glia correspond to the glial layer with the closest association to NSCs and progeny when larval NSCs start cycling. They are generated during embryogenesis, from the activity of specific NSC lineages (NB6-4, NB6-4T and NB7-4) (Ito et al., 1995; Awasaki et al., 2008; Beckervordersandforth et al., 2008). There, their functions and structure are little known. At larval hatching, when NSCs are still quiescent, the dual structure of the cortex glia around NSC lineages is not present, and cortex glia rather appear as a discontinuous meshwork with no individual encasing of NSCs. Cortex glia structure indeed remodels during larval life (Pereanu et al., 2005). Recent works have started elucidating the morphogenetic processes supporting the progressive formation of this elaborate glial structure in later larval stages. This process can be divided in three main steps (Spéder and Brand, 2018): expansion, during which the cortex glia grow parallel to NSC reactivation and the chamber is still not formed; chamber formation, when individual NSC are encased around the time they perform their first division; and extension, during which the cortex glia keep adapting to the growth of NSC lineages while maintaining their chamber organization. The expansion phase corresponds to a massive production of cortex glia membranes and, like NSC reactivation, is nutritionally-regulated (Spéder and Brand, 2018; Yuan et al., 2020). More precisely, the production and secretion of dILPs in the subperineurial glia leads to the autonomous activation of the conserved PI3K-Akt signalling pathway in the cortex glia. Starvation, preventing dIlps production and release from the subperineurial glia, and blocking the activation of the PI3K/Akt pathway in the cortex glia all prevent cortex glia expansion. Interestingly, PI3K/Akt-dependent growth of the cortex glia during the expansion phase itself seems to promote NSC reactivation, pinpointing some coordination between the two cell types (Yuan et al., 2020). The nutrition-fueled cellular growth of the CG is supported by a number of proliferative strategies (Pereanu et al., 2005; Avet-Rochex et al., 2012; Rujano et al., 2022) to match its extent. CG first undergo endoreplicative cycles, seemingly both endocyles (an alternance of S and G2 phases) and endomitosis (displaying some mitotic hallmarks but without nuclear division), leading to their polyploidy. CG growth appears mostly autonomous, at least partly regulated by the homeodomain transcription factor Cut (Yadav et al., 2023), yet with some moderate input from NSC reactivation (Spéder and Brand, 2018; Yuan et al., 2020). In contrast, chamber formation and individual NSC encasing are highly dependent of NSC reactivation, and timely-correlated with NSC re-entry into the cell cycle (Spéder and Brand, 2018). The cues sent by reactivating NSCs to recruit cortex glia membrane and trigger their encasing are so far unknown, and likely to combine signalling and adhesion processes. Interestingly, a NSC (Pvf ligand secretion) to cortex glia (Pvr receptor signalling) interaction is also crucial for proper morphogenesis of the cortex glia (Read, 2018), and might be a good candidate if the temporal window of its requirement fits. Other cortex glia to NSC physical interactions are also set up during development through Neurexin to Wrapper interaction (Banach-Latapy et al., 2023), and septate junctions have been detected between the two cell types (Pereanu et al., 2005).

Chamber formation is a critical morphogenetic event matching a tipping point in NSC behaviour. First, reactivation is complete, and active proliferation by asymmetric division starts. NSCs are also becoming independent from nutrition-induced PI3K/Akt signalling and their proliferation is rather sustained by the Alk pathway, whose ligand Jelly Belly appears produced by the now fully enwrapping cortex glia (Cheng et al., 2011). Their access to external factor also changes, as their encasing by the cortex glia inserts an extra layer between NSCs and the blood-brain barrier/the environment. Indeed this cortex glia layer appears to provide some permeability barrier for solute (Yuan et al., 2020) and, while it needs to be demonstrated, is likely to regulate the passage of many signalling molecules. In this light, it is striking that NSC dependence on subperineurial glia-secreted dIlps ends when the cortex glia chamber forms (Cheng et al., 2011; Spéder and Brand, 2018).

Cortex glia behaviour also seems to change after chamber formation, during the extension phase. While they still need to grow to accommodate the extensive generation of newborn neurons by actively cycling NSCs, endoreplication slowly dwindles down, and another replicative strategy, acytokinetic miosis, takes place (Rujano et al., 2022; Yadav et al., 2023). Acytokinetic mitosis leads to nuclear division without cellular separation, thus forming multinucleated, syncytial cells. Much like endoreplication, this strategy ensures that one cortex glia cell will be equipped with several genome copies supporting important protein production and cellular growth, and that such growth will not disrupt cellular architecture.

An important feature of cortex glia organization is indeed the combination of cell size and cell arrangement. After expansion and from the time of encasing, one cortex glia cell spans several NSCs, while forming a membranous chamber around each (Rujano et al., 2022). These cellular units thus physically partition the NSC population in subgroups, while organising themselves as a cellular mosaic tiling the whole CNS. These NSC subgroups varies between individuals and as such do not seem to be predetermined. Whether the process is fully stochastic remains to be assessed. In addition to the population (network) and the individual (chamber) scales, the discovery of such organization identified a third, regional (unit) scale of the cortex glia niche. From early larval stage, cortex glia cells thus appear to grow, enwrapping neighbouring NSCs, until they meet each other and set cellular boundaries. The picture is still more complex, as cortex glia units can undergo cellular fusion, sharing membrane, cytoplasm, and organelle’s compartments (Figure 3, bubble 5). These fusion events vary in time and space. Even more strikingly, they appear transient, as compartments once connected can lose this ability to exchange (Rujano et al., 2022). The existence of cellular fusion between CG units, combined with its variability and transientness, results in changing boundaries between the units, making the regional structure of the niche plastic around NSCs and progeny. What regulates the time, place, frequency and duration of cell fusion in the cortex glia remains a mystery.

#### 5.3.4 Trachea

The *Drosophila* tracheal system comprises a complex network of epithelial tubes responsible for transporting oxygen throughout the body and generated through a series of complex morphogenetic events (described in (Hartenstein, 1993) and reviewed in (Ehrhardt et al., 2022)). This intricate structure originates from 20 ectodermal cell clusters, each composed of approximately 80 cells, and visible very early during development (5–6 h after egg laying). These clusters undergo a process of invagination, forming sac-like epithelial structures, one in each segment. Primary, secondary and terminal branches sequentially bud from these sacs, contributing to the formation of the tracheal network through cell migration and fusion (Samakovlis et al., 1996; Sutherland et al., 1996). Ganglionic branches (represented in Figure 3) target the ventral nerve cord and cerebral branches target the central brain (Hartenstein, 1993; Pereanu et al., 2007). A ganglionic branch (one per segment) is a primary epithelial tube made by seven cells (Samakovlis et al., 1996). During budding and elongation, a single cell at the tip of the emerging structure leads the migratory process towards the CNS. This process starts during embryogenesis around 10 h after egg laying and is guided by the presence of clusters of epidermal cells expressing *branchless,* a member of the Fibroblast Growth Factor family (Sutherland et al., 1996). Within the next two hours, the leading cell at the tip of the ganglionic branch reaches, migrates along and finally enters the ventral nerve cord laterally and ventrally (Samakovlis et al., 1996; Englund et al., 1999). By the beginning of the larval stage, a ring-like rudimentary tracheal plexus (much like the mammalian perineural vascular plexus) is set up around the neuropile by the invasion, extension/migration and fusion of segmental ganglionic branches. From there, this plexus will grow and branch throughout larval stages to form a complex network mostly localized close to the neuropile (Pereanu et al., 2007; Baccino-Calace et al., 2020). Their growth appears constrained by glial cells (Pereanu et al., 2007) and involves some proliferative mechanisms at late stages (Rao et al., 2015). Little is known about the coordination between trachea growth, function and interplay with other cell types of the niche. One common sensitivity is to nutrition-induced PI3K/Akt signalling at early stage, which fuels tracheal growth. In turn, tracheal growth seems to favour NSC reactivation (Yuan et al., 2020). Whether this role is linked to the supply of oxygen or of other signalling factors is not known. Interestingly, the distance between the tracheal network and NSCs increases during larval stage along with NSC lineage growth (Bailey et al., 2015).

#### 5.3.5 The fat body: an example of a distant niche

The fat body is a crucial player in NSC reactivation, secreting nutrition-responsive factors, yet unknown, which reach the CNS and trigger insulin signalling from the glial cells (Britton and Edgar, 1998; Chell and Brand, 2010; Sousa-Nunes et al., 2011; Spéder and Brand, 2014). Fat body development (reviewed in (Zheng et al., 2016; Li et al., 2019; Parra-Peralbo et al., 2021)) starts with the progressive apparition of mesodermal cell clusters between embryonic stages 10-14 (5–10 h after egg laying). These fat body primordia undergo mitosis to reach around 2,200 cells and shortly afterwards at stage 16 (13–16 h after egg laying) fuse to generate one continuous monolayer of fat cells. These cells keep growing through endoreplication, until entering quiescence in late embryogenesis. Fat cells will resume endoreplication around 8 h after larval hatching, paralleling the timing of NSC reactivation, and grow throughout larval stages without a change in number, while storing various nutrients.

## 6 Conclusion

### 6.1 Take home messages across all model systems

Niche morphogenesis is a coordinated process between different cell types and acellular components to ensure that the needs of the tissue to develop and acquire specialized functions are met in the right time and place. The timing of growth, adhesion and inter-cellular communication will generate a specific, yet dynamic, niche architecture, which in turn is a powerful means to regulate the reach of signalling.

The scales of both niche morphogenesis and niche architecture are multifold. Niche morphogenesis spans time (from early embryonic development to early post-natal growth, and even adulthood) and tissues (neural, hematopoietic, and immune compartments). Niche architecture is built on cell-to-cell adhesions (ECM, adherens junctions, tight junctions, surface immunoglobulins, gap junctions), which will act to generate local structures (*e.g.*, astrocyte/blood vessel/RGC basal process unit; cortex glia chamber) and signalling (*e.g.*, primary cilia of RGCs; ECM/integrin signalling), but which also can reach to farther regions of the tissue (*e.g.*, gap junction coupling in the subperineurial glia; attachment of RGCs’ basal process to blood vessels; ECM as a mechanical constraint). Some of these molecular and cellular processes can also be regional (*e.g.*, ependymal pinwheel structure grouping few RGCs together; mosaic of cortex glia), producing “mini-niches” within the niche.

### 6.2 Where the neurogenic niche field should go next

Stem cell niches have emerged as powerful and plastic regulators of stem cell behaviour. The neurogenic niche is no exception yet distinguishes itself by its especially heterogeneous and complex composition, the inclusion of components with unique role at the organism level (*i.e.*, the blood-brain barrier) and its multiscale architecture.

While the field has made critical progress in the signalling aspect of the neurogenic niche, and despite the growing understanding that mechanical cues and physical constraints have powerful implications, little is still known on niche architecture.

The first question which has been explored in recent years is how the niche forms and acquired its “final” (mature) architecture (at least in homeostatic conditions), since such understanding is a prerequisite to its manipulation for probing its function. Deciphering niche formation includes the identification of the morphogenetic processes at play, their cellular and molecular players, and the adhesive properties eventually set in place. The mouse and the fruit fly have been leading this aspect, with different focuses, and with still much left to comprehend. Niche morphogenesis is pretty much unknown in the CNS of other organisms, and one of the further challenges of the field is to bring this comparative understanding to the table.

Nearly all is left to achieve on the other aspects of niche architecture, covering scales, rules, and functions. Our own perception of the neurogenic niche, and its relationship with other fields of biological research, would suggest concentrating on the following questions.

1. Interplay between architecture and signalling, including physical/mechanical cues both as a signal *per se* and as a modulator of signalling range
2. Relationship between local, regional and tissue scales, especially in relationship with the cellular coordination or independence within the NSPC population
3. Plasticity in response to time (from development to ageing), signals (metabolic status), and conditions (pathologies)

The answers to all these questions bid exciting times for stem cell biology, converging with other outstanding questions in the field touching their regulation under stress and their double-edged potential for curative (*e.g.*, regeneration) and pathological (*e.g.*, tumourigenesis) outcomes.
